# Metabolic Profiling in Association with Vascular Endothelial Cell Dysfunction Following Non-Toxic Cadmium Exposure

**DOI:** 10.3390/ijms18091905

**Published:** 2017-09-05

**Authors:** Qiuan Zhong, Xiaofei Li, Qingjiao Nong, Baoyu Mao, Xue Pan

**Affiliations:** 1 Guangxi Colleges and Universities Key Laboratory of Prevention and Control of Highly Prevalent Diseases, Guangxi Medical University School of Public Health, Nanning 530021, China; lixiaofeifudan@gmail.com; 2 Department of Epidemiology, Guangxi Medical University School of Public Health, Nanning 530021, China; qjnong@gmail.com (Q.N.); maobaoyu@stu.gxmu.edu.cn (B.M.); panxue@stu.gxmu.edu.cn (X.P.)

**Keywords:** cadmium, nitric oxide, vascular endothelial function, metabolomics, metabolic pathway

## Abstract

This study aimed to determine the metabolic profile of non-toxic cadmium (Cd)-induced dysfunctional endothelial cells using human umbilical vein endothelial cells (HUVECs). HUVECs (*n* = 6 per group) were treated with 0, 1, 5, or 10 μM cadmium chloride (CdCl_2_) for 48 h. Cell phenotypes, including nitric oxide (NO) production, the inflammatory response, and oxidative stress, were evaluated in Cd-exposed and control HUVECs. Cd-exposed and control HUVECs were analysed using gas chromatography time-of-flight/mass spectrometry. Compared to control HUVECs, Cd-exposed HUVECs were dysfunctional, exhibiting decreased NO production, a proinflammatory state, and non-significant oxidative stress. Further metabolic profiling revealed 24 significantly-altered metabolites in the dysfunctional endothelial cells. The significantly-altered metabolites were involved in the impaired tricarboxylic acid (TCA) cycle, activated pyruvate metabolism, up-regulated glucogenic amino acid metabolism, and increased pyrimidine metabolism. The current metabolic findings further suggest that the metabolic changes linked to TCA cycle dysfunction, glycosylation of the hexosamine biosynthesis pathway (HBP), and compensatory responses to genomic instability and energy deficiency may be generally associated with dysfunctional phenotypes, characterized by decreased NO production, a proinflammatory state, and non-significant oxidative stress, in endothelial cells following non-toxic Cd exposure.

## 1. Introduction

Endothelial-derived nitric oxide (NO), the most important vasodilator molecule, is a key regulator of endothelial homeostasis. Loss of NO bioavailability induced by decreased synthesis or increased degradation has been well recognized as a crucial factor for vascular endothelial dysfunction, which contributes to the pathological progression of atherosclerotic cardiovascular diseases [1,2,3,4]. Cadmium (Cd), a toxic heavy metal even at low levels, has been clearly shown to reduce NO production in vascular endothelial cells in vitro, and subsequently promote the early development of atherogenesis in vivo [5,6,7]. Furthermore, growing population-based evidence supports that long-term Cd exposure, even at low levels, is a novel risk factor for various cardiovascular outcomes [8,9,10,11,12]. The underlying pathological mechanisms point towards the onset of atherogenesis, showing that NO-related endothelial dysfunction could be induced by the toxic effects of Cd.

Indeed, impairment of NO bioavailability in endothelial cells indicates a systemic condition, including dysfunctional phenotypes across not only the inhibition of NO production, but also a proinflammatory state and prothrombic formation [1,3,13]. Metabolomics is an efficient approach for the detection and dynamic profiling of endogenous metabolites from biospecimens, especially regarding systemic and complicated phenotypes, thereby discovering relevant biomarkers or mechanisms. Until now, metabolic responses to chronic Cd exposure have been used to identify early biomarkers of health effects in human urine [14,15] and to assess adverse effects on liver function in vivo [16]. However, less is known about the metabolic alterations of vascular endothelial cells towards endothelial cell dysfunction phenotypes induced by non-toxic Cd concentrations.

In this study, we aimed to characterize endothelial dysfunction phenotypes, including NO production, inflammatory response and oxidative stress, in human umbilical vein endothelial cells (HUVECs) treated with low-dose Cd and to identify endogenous metabolic profiles and pathways, by using non-targeted gas chromatography time-of-flight/mass spectrometry (GC-TOF/MS), a metabolomics technology with high resolution and detection sensitivity [17,18]; additionally, we evaluated potential metabolic mechanisms associated with vascular endothelial cell dysfunction.

## 2. Results

### 2.1. HUVEC Growth and Tube Formation Ability Induced by Cd Exposure

**Figure 1:**
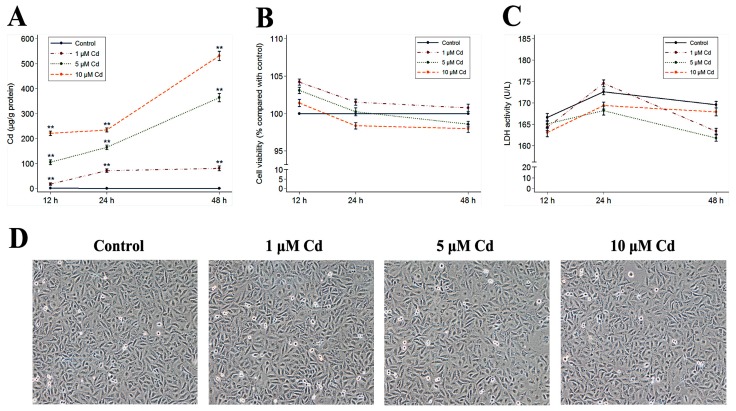
Effects of Cd on HUVEC growth. Data on intracellular Cd content (**A**), cell viability (**B**), and LDH activity (**C**) in HUVECs treated with 0, 1, 5, or 10 μM Cd for 12 h, 24 h, or 48 h. Data on the Cd content (**A**) and the LDH activity (**C**) represent mean ± SEM (*n* = 6), data on the cell viability (**B**) represent mean ± SEM from three independent experiments (*n* = 8 per experiment). The asterisks indicate statistically significant differences (** *p* < 0.001, one-way analysis of variance (ANOVA) with Bonferroni post hoc test) compared to the control values for the corresponding incubation times. The control or Cd-exposed cell morphology for 48 h (**D**) was observed under a phase-contrast microscope at 10× magnification.

Clearly, elevated levels of intracellular Cd were observed with increased incubation duration with 1, 5, and 10 μM of cadmium chloride (CdCl_2_) (Figure 1A). Compared to control groups, different doses of CdCl_2_ did not significantly affect relative cell viability (Figure 1B), lactate dehydrogenase (LDH) activity (Figure 1C), or typical cell cobblestone morphology (Figure 1D) for 48 h of Cd exposure, suggesting that long-term exposure to low-dose Cd between 1 and 10 μM does not substantially affect HUVEC growth reflected in cell proliferation, membrane integrity, or morphology.

**Figure 2:**
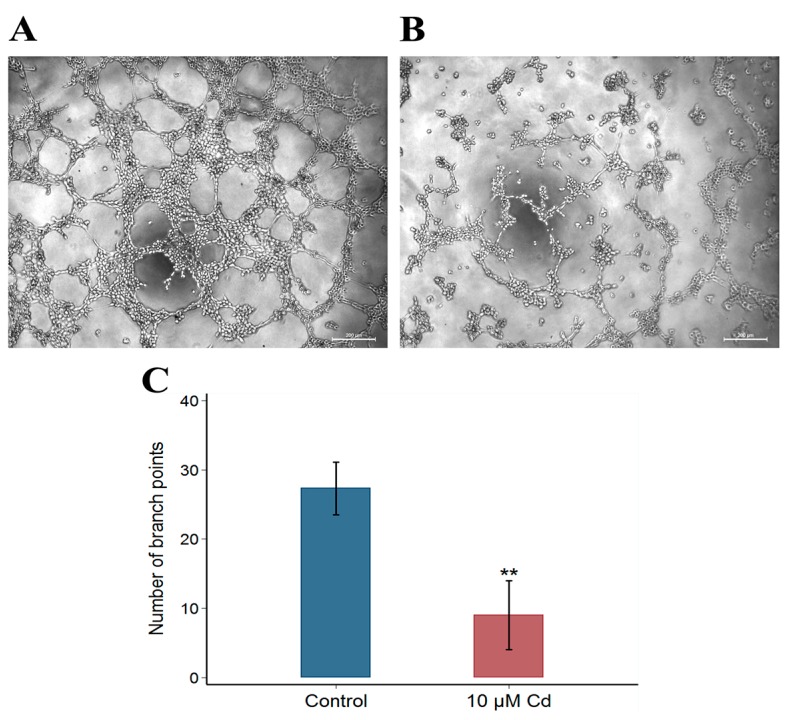
Effects of Cd on HUVEC tube formation. Control cells (**A**) were not treated with Cd, and cells (**B**) were treated with 10 μM Cd for 48 h. Both images were observed under a phase-contrast microscope at 50× magnification. Data on the number of branch points (**C**) represent mean ± SEM from three independent experiments. The asterisk indicates statistically significant differences (** *p* < 0.001, Student’s *t*-test) compared to the control value. Scale bars = 200 μm.

As shown in Figure 2, the number of branch points were significantly decreased in Cd-exposed group compared to control group (*p* < 0.001), indicating an inhibition of tube formation ability in HUVECs treated with 10 μM CdCl_2_.

### 2.2. Ultrastructure of HUVECs

**Figure 3:**
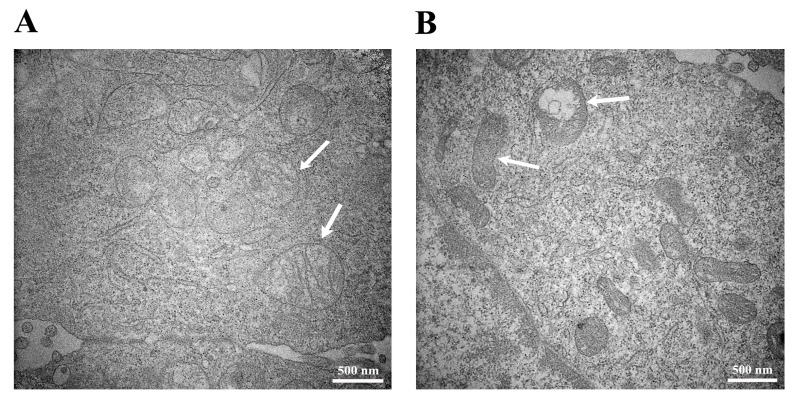
TEM image of HUVECs. Cells were not treated with Cd as a control (**A**) or were treated with 10 μM Cd for 48 h as the Cd-exposed sample (**B**). Both the control and Cd-exposed samples were observed at 40,000× magnification and 100 KV. The arrows indicate the oval or irregular round mitochondria in (**A**) and the elongated or vacuolated mitochondria in (**B**).

Transmission electron microscopy (TEM) images revealed that organelle morphology was not significantly altered in the Cd-exposed HUVECs compared to the control cells, except in the mitochondria. The mitochondria in the control samples exhibited the typical oval or irregular round shape (Figure 3A). In contrast, few mitochondria displayed an elongated shape or vacuolated in the Cd-exposed samples (Figure 3B).

### 2.3. HUVEC Phenotype Induced by Cd Exposure

**Figure 4:**
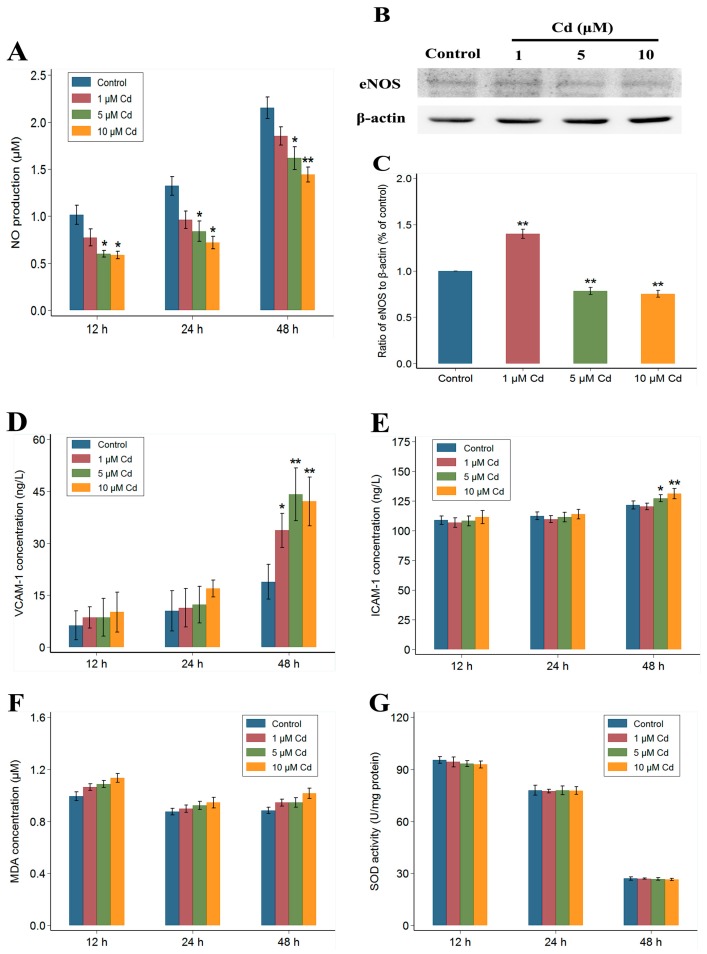
Effects of Cd on NO production (**A**), eNOS expression (**B**,**C**), VCAM-1 and ICAM-1 concentrations (**D**,**E**), MDA concentrations (**F**), and SOD activity (**G**) in HUVECs. HUVECs (**A**,**D**–**G**) were treated with 0, 1, 5, or 10 μM Cd for 12, 24, or 48, and HUVECs (**B**) were treated with 0, 1, 5, or 10 μM Cd for 48 h. Data (**A**,**D**–**G**) represent mean ± SEM (*n* = 6), and data on the eNOS expression (**C**) represent mean ± SEM from three independent experiments. The asterisks indicate statistically significant differences (* *p* < 0.05, ** *p* < 0.001, one-way ANOVA with Bonferroni post hoc test) compared to the control values for the corresponding incubation times.

**Figure 5:**
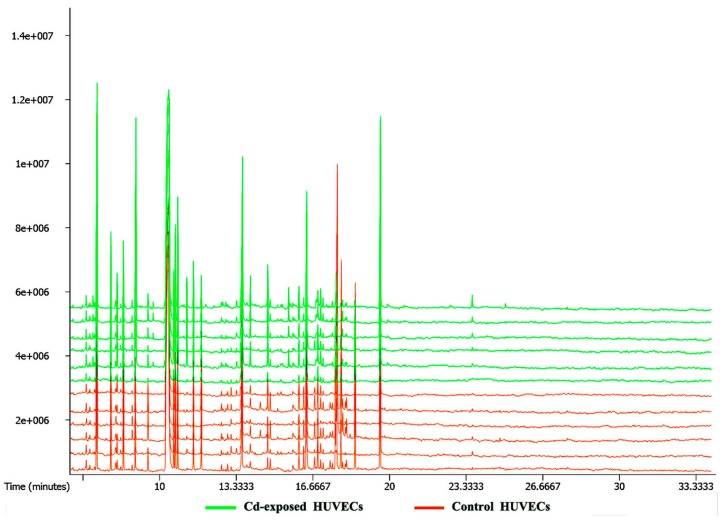
Typical GC-TOF/MS TICs of Cd-exposed HUVECs and control HUVECs. HUVECs were treated with 0 or 10 μM Cd for 48 h. The vertical axis represents the relative mass abundance, and the horizontal axis represents the retention time (RT).

As seen in Figure 4A, 5 and 10 μM CdCl_2_ significantly inhibited NO production in HUVECs compared to controls (*p* < 0.05); however, a non-significant inhibition of NO production was observed following 1 μM CdCl_2_ treatment. Additionally, 5 and 10 μM CdCl_2_ treatment for 48 h significantly decreased the expression of eNOS in HUVECs compared to control (*p* < 0.001) (Figure 4B,C). Furthermore, concentrations of vascular cell adhesion molecular 1 (VCAM-1) and intercellular adhesion molecule 1 (ICAM-1) were significantly increased following 5 and 10 μM CdCl_2_ treatment at 48 h (*p* < 0.05) (Figure 4D,E), indicating a proinflammatory state in Cd-exposed HUVECs. However, the evaluation of oxidative stress, comparing various Cd-treated with control HUVECs, revealed non-significant increases in malondialdehyde (MDA) concentrations and non-significant changes in superoxide dismutase (SOD) activity for the Cd exposure duration regardless of 12, 24, or 48 h (Figure 4F,G).

### 2.4. Metabolic Profiling of GC-TOF/MS

Typical GC-TOF/MS total ion chromatograms (TICs) of Cd-exposed and control samples are shown in Figure 5. In general, Cd-exposed and control samples could be distinguished by their relative peak areas. A total of 474 raw peaks were originally detected in the TICs, and 433 valid peaks remained after missing value imputation and data denoising. A total of 206 compounds were identified from the 433 valid peaks, including amino acids, organic acids, carbohydrates, and nucleosides.

### 2.5. Differential Metabolites between the Cd-Exposed and Control Groups

**Figure 6:**
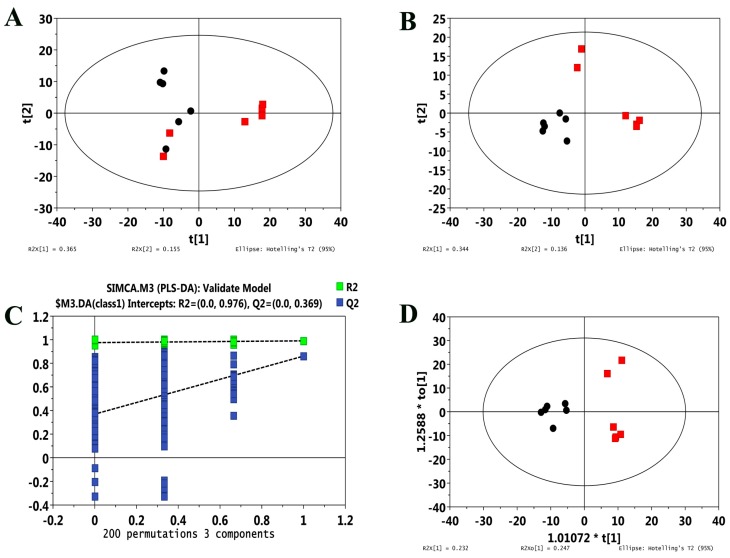
PCA score plot (**A**), score plot (**B**) and permutation test plot (**C**) of the PLS-DA, and the OPLS-DA score plot (**D**) of the GC-TOF/MS metabolic profiles from Cd-exposed HUVECs and control HUVECs. Red squares (**A**,**B**,**D**) represent Cd-exposed HUVECs, and black circles (**A**,**B**,**D**) represent control HUVECs.

As shown in Figure 6A, the metabolic profiles of HUVECs were not significantly different between the Cd-exposed and control groups in the score plot of the principal component analysis (PCA) model, which presented all of the samples inside the 95% Hotelling T^2^ ellipse. For the model quality in discriminating metabolites between Cd-exposed and control samples, robustness, as well as goodness of fit and prediction, were suggested by the score plot and the permutation test plot of the partial least-squares discriminant analysis (PLS-DA) model (Figure 6B,C). The R^2^Y and Q^2^Y values were 0.99 and 0.861 for the seven-fold cross-validation test, respectively; the R^2^ and Q^2^ the intercept values were 0.975 and 0.377 for the permutation test, respectively. Furthermore, a clear discrimination was observed between the Cd-exposed and control groups from the score plot of the orthogonal projections to latent structures discriminant analysis (OPLS-DA) model (Figure 6D), suggesting that specific metabolites responded to Cd exposure were significantly different compared to control samples.

As listed in Table 1, a total of 24 reliable metabolites with a variable importance in the projection (VIP) value >1 were identified by the OPLS-DA model to be significantly different in Cd-exposed HUVECs compared to controls. Compared to the control group, 20 differential metabolites with a fold change value >1 were up-regulated in the Cd-exposed group, including seven amino acids, three pyrimidines and derivatives, two purines and derivatives, four carbohydrates and carbohydrate conjugates, two carboxylic acids and derivatives, one alkanolamine, and one fatty acid and conjugate. A total of four metabolites with a fold change value <1 were down-regulated, including three carboxylic acids and derivatives, as well as one carbohydrate and carbohydrate conjugate.

### 2.6. Metabolic Pathways Related to Differential Metabolites

**Figure 7:**
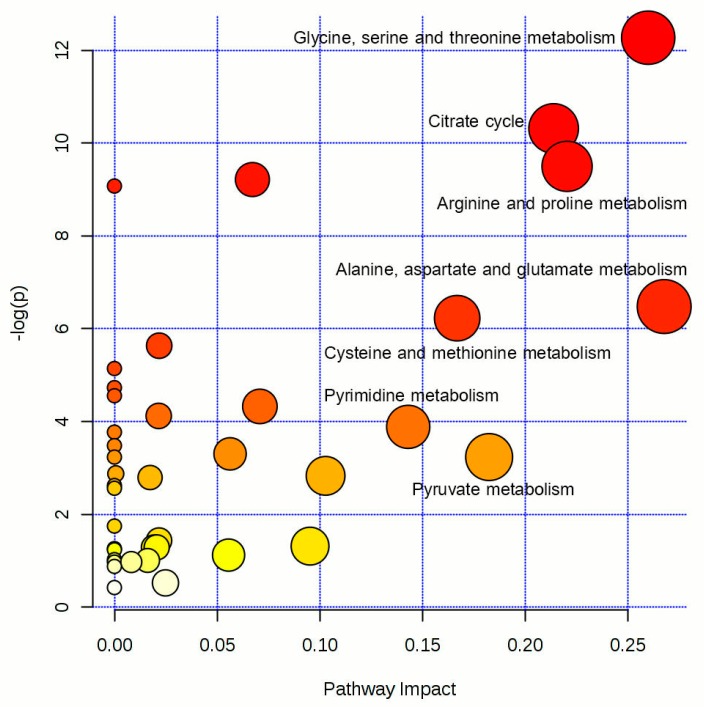
The disturbed metabolic pathways in Cd-exposed HUVECs compared to control HUVECs.

Among 24 differential metabolites, seven significantly-altered metabolic pathways were revealed from the pathway analysis, including glycine, serine, and threonine metabolism, as well as the tricarboxylic acid (TCA) cycle, arginine and proline metabolism, alanine, aspartate, and glutamate metabolism, cysteine, and methionine metabolism, pyrimidine metabolism, and pyruvate metabolism (Figure 7). The seven significantly-altered pathways met the conditions of a *p*-value of enrichment analysis <0.05 and a pathway impact value >0.1. The differential metabolites involved in the significantly-altered metabolic pathways are shown in Table 2.

## 3. Discussion

As an important aspect of systems biology, metabolomic findings are vital for the elucidation of mechanisms involved in cardiotoxicity, especially that caused by exogenous chemicals. In this study, our results clearly showed that although the growth of endothelial cells was not affected, the tube formation ability of endothelial cells was substantially impaired by long-term exposure to low-dose Cd. In this context of endothelial cell dysfunction, as the predominant phenotype in HUVECs, deceased NO production was accompanied by a proinflammatory state and non-significant oxidative stress. These cell dysfunction phenotypes were associated with significant changes in 24 endogenous metabolites, which were involved in seven altered metabolic pathways in the present metabolomics analysis.

Environmental Cd pollution, generally at non-toxic levels, has been a global issue for public health [19]. To determine the effects of environmental Cd toxicity on metabolism, previous metabolic profiling study using human urine have identified six metabolites involved in mitochondrial metabolism or one-carbon metabolism by high-resolution ^1^H nuclear magnetic resonance (NMR) spectroscopy [14]. Another study identified 27 metabolites related to carbohydrate, amino acid, bone and intestinal flora metabolism, as well as the TCA cycle by GC-MS [15]. However, direct studies of the metabolomics in vascular endothelial cell dysfunction have, to our knowledge, been poorly lacking regarding the effects of Cd toxicity. With this background, although there is a growing awareness that vascular endothelium is a promising target for preventive or therapeutic strategies in atherogenesis [20], it is still limited to reversing the progression of atherosclerosis, fundamentally, partly owing to lack of a metabolic understanding of endothelial dysfunction phenotypes in the setting of specific risk factors.

In the current study, our TEM analyses indicated the endothelial mitochondria were a target organelle for Cd toxicity in accordance with previous reports [21,22], which confirmed a direct toxic effect of Cd on endothelial mitochondria, resulting in activation of the mitochondrial death pathway or signals. As an organelle containing an independent genome, endothelial mitochondria play a primary role in response to environmental stimuli via signal transduction, which is essential for maintaining the endothelial function [23]. Furthermore, it has been found that mitochondrial dysfunction is closely associated with the progression of atherogenesis in human atherosclerotic plaques [24].

In parallel with the TEM observations, the metabolic findings clearly implicated an impairment or dysfunction of mitochondria in Cd-exposed HUVECs, based on the following metabolic profiles. First, among the 24 altered metabolites, three organic acids, including L-malic acid, fumaric acid, and citric acid, which are key intermediates in the TCA cycle in mitochondria [25], were down-regulated in Cd-exposed HUVECs, indicating a dysfunction of the TCA cycle. Similar results of the TCA cycle were found in mouse renal proximal tubules incubated in vitro with low-dose Cd [26]. Second, all of the up-regulated amino acids in Cd-exposed HUVECs belong to glucogenic amino acids. The TCA cycle is a common metabolic pathway for carbohydrate, fat, and protein metabolism in the mitochondria of eukaryotic cells. Therefore, TCA cycle dysfunction may further cause an increase in glucogenic amino acids, since glucogenic amino acids (e.g., aspartic acid, glycine, and cysteine) are generally catabolized into different intermediates, such as pyruvate, oxaloacetate, and alpha-ketoglutarate, that enter the TCA cycle to maintain normal metabolic function [27]. Third, the metabolic alterations showed a genomic instability in Cd-exposed HUVECs. Compared to control, Cd-exposed HUVECs had higher levels of pyrimidines (uracil and thymine) and purines (guanine and hypoxanthine), indicating genomic instability, which could generally be induced by the toxic effects of Cd even at non-cytotoxic concentrations [28,29,30,31]. Similarly, the presence of oxidative DNA lesions was implicated by elevated levels of ribonolactone, a major type of oxidized abasic site, in Cd-exposed HUVECs, since ribonolactone levels are correlated with reductions in the repair capacity of DNA in mammalian cells [32,33,34]. Given more mitochondrial DNA than nuclear DNA damage in response to Cd toxicity [35,36], the genomic instability of endothelial cells, thus, may derive from the impaired mitochondria, as well as nuclear DNA alterations.

Generally, endothelial-derived NO is synthesized from l-arginine, oxygen and nicotinamide adenine dinucleotide phosphate (NADPH) by eNOS. Low-level CdCl_2_ exposure could significantly attenuate vascular endothelial eNOS activity and further inhibit NO production, which has been well confirmed in vitro or in vivo [37,38,39]. The underlying mechanisms for Cd-induced NO production inhibition in endothelial cells have been partly elucidated, including blocking eNOS phosphorylation, attenuating eNOS expression, and blocking eNOS translocation [37,38,39]. Notably, previous studies in vivo have shown an eNOS-dependent mitochondrial biogenesis and function in caloric restriction or exercise training models [40,41]. In view of these facts, the decreased eNOS activity might impair the mitochondrial function of endothelial cells in this study. On the other hand, a recent study indicated that the disturbance of mitochondrial calcium homeostasis, a characteristic of Cd-induced cytotoxicity in vitro [21], substantially influenced NO production via regulating eNOS activity in endothelial cells [42]. Therefore, the possibility that the impaired mitochondria could partly inhibit endothelial-derived NO production cannot be ruled out in this study. In brief, it may exist a positive loop between decreased eNOS activity and impaired mitochondria in Cd-exposed endothelial cells, suggesting that the metabolic alterations mentioned earlier may be triggered by not only the impaired mitochondria per se, but also the inhibited production of NO.

For the inflammatory response, low to modest levels of CdCl_2_ (40-80 μM) significantly increased interleukin (IL)-1β production in HUVECs [43], and non-toxic Cd concentrations could substantially induce the expression of VCAM-1 and ICAM-1 via activating the related protein kinase and reactive oxygen species (ROS) in endothelial cells [44,45], as well as the expression of VCAM-1 in ApoE*−*/*−* mice [7]. Similarly, our findings in VCAM-1 and ICAM-1, indicating an active state of proinflammation, were consistent with those from previous studies of the inflammatory response. In the current altered metabolites, ethanolamine (18.39-fold higher in Cd-exposed HUVECs) is the second most abundant substrate for phospholipids in biological membranes. Phospholipids and their oxidized products have been proven to contribute to proinflammatory states in endothelial cells [46,47]. In this study, therefore the increased VCAM-1 or ICAM-1, a transmembrane protein, may be associated with overproduction of ethanolamine. In addition, it is worth noting that levels of *N*-acetylmannosamine (ManNAc), compared to control, markedly increased (169.09-fold) in Cd-exposed HUVECs. ManNAc, a hexosamine monosaccharide, is a by-product of uridine diphosphate *N*-acetylglucosamine (UDP-GlcNAc), which is a key intermediate for protein glycosylation in the hexosamine biosynthesis pathway (HBP) [48]. In view of this, the markedly increased ManNAc may indicate an enhanced glycosylation reaction from the HBP in Cd-exposed HUVECs. Of note, previous evidence in vitro implicated that *N*-glycosylation of protein adhesion molecules, including VCAM-1 and ICAM-1, differentially regulated leukocyte recruitment in heterogeneous endothelial cells in response to proinflammatory stimuli, in addition to upregulation of protein adhesion molecule expression [49]. Whereas decreased expression of ICAM-1 and impaired inflammatory response were found in glycosylation-deficient mice [50]. Considering these findings, the present study suggests that specific glycosylation inhibition may be a potential option against non-toxic Cd-induced inflammatory responses. Interestingly, inhibition of COX-2 *N*-glycosylation has been proven to effectively raise an anti-inflammatory effect in collagen-induced arthritic mice [51].

As an oxidation state metal, Cd is widely found to elevate cellular oxidative stress via modification of thiol protein or inducing apoptosis [52,53]. However, we indicated a non-significant oxidative stress with MDA and SOD activity, which generally represent lipid peroxidation and antioxidant defences, respectively, in the current HUVECs exposed to chronic low-dose Cd. With respect to the discrepancy, a study has proposed that high concentrations of Cd lead to cytotoxicity, while a compensatory protective response to lower concentrations of Cd in endothelial cells [54].

Among the altered amino acids, l-homoserine was markedly elevated (204.37-fold) in Cd-exposed HUVECs. l-homoserine, a non-proteinogenic amino acid synthesized from aspartic acid, is an intermediate in the biosynthesis of methionine and threonine in microorganisms and plants [55]. The l-homoserine and its involved pathways (glycine, serine and threonine; cysteine and methionine) are pivotal elements for one-carbon metabolism, which is crucial for redox balance and nucleotide synthesis [56,57]. The markedly increased l-homoserine, thus, indirectly implicated a self-repairing status by one-carbon metabolism. Additionally, although it existed genomic instability mentioned earlier, there was significantly higher orotic acid (10.53-fold), an essential intermediate of pyrimidine de novo synthesis, in Cd-exposed HUVECs, also indicating an improvement of pyrimidine synthesis [58]. Simultaneously, there are metabolic cues for maintaining cellular growth through energy offset. In this study, levels of pyruvic acid (11.33-fold) significantly increased in Cd-exposed HUVECs. Pyruvic acid, the simplest of the alpha-keto acids, is the end product of glycolysis [59], which generally converts glucose into pyruvic acid while generating adenosine triphosphate (ATP) and nicotinamide adenine dinucleotide (NADH) in the cytoplasm. Given that the amounts of ATP were decreased by the impaired TCA cycle in mitochondria, the glycolysis was coordinately enhanced, as indicated by the increased pyruvic acid and the activated pyruvate metabolism [60], which constituted a compensatory response to ATP deficiency for cellular growth. Meanwhile, metabolic intermediates of enhanced glycolysis would fuel side pathways, such as the pentose phosphate pathway (PPP) [61], subsequently generating NADPH that contributes to scavenging ROS [48,62]. Therefore, these above metabolic alterations may be attributable to the compensatory mechanisms for genomic stability and energy demand, finally resulting in non-significant oxidative stress, as well as salvage cell growth in this study.

Taken together, the current metabolic profiles seem to depict a dynamic mechanism in damage, repairing, and salvage for the vascular endothelial cells following non-toxic Cd exposure. As a result, although the oxidative stress was ameliorated, decreased NO production and a proinflammatory state substantially occurred in dysfunctional endothelial cells. Our metabolic findings further suggest that restoring the TCA cycle and regulating glycosylation of the HBP, rather than antioxidant supplements, may be the prior strategies in prevention or treatment for the early stage of non-toxic Cd-induced atherogenesis, via reversing endothelial dysfunction with expected manifestations in NO production and inflammatory response. Further studies are needed to accurately connect the metabolic alterations to specific endothelial phenotype for verification of these hypotheses.

There are several limitations in this study. First, we may not be able to efficiently detect less volatile and strongly polar compounds due to the limitations of GC-MS [63], thus resulting in biased metabolic profiles of endothelial cells. Moreover, although we have identified the differential metabolites and significant metabolic pathways in the current study, metabolic perturbations may not yet be accurately associated with each dysfunctional phenotype in endothelial cells. This awkward situation needs to be met with targeted designs for specific phenotype in future studies. Finally, as a complex and dynamic response to environmental stimuli, either of inflammatory response and oxidative stress is involved in not only multiple organelles, but also different signalling mechanisms in cells. Perhaps these phenotypes cannot be comprehensively evaluated using only a combination of VCAM-1, ICAM-1, MDA, and SOD activity in the present study, thus, our findings should be interpreted with caution.

## 4. Materials and Methods

### 4.1. Materials

Cadmium chloride (CdCl_2_) was purchased from Tianjin Damao Chemical Reagent Co., Ltd. (Tianjin, China). Standard Cd solutions of 1000 μg/mL were obtained commercially from the National Institute of Metrology, China (Beijing, China). A nitric oxide colourimetric assay kit, malondialdehyde (MDA) assay kit, and CCK-8 cell viability assay kit were obtained from GenMed Scientifics (Shanghai, China). A human intercellular adhesion molecule 1 (ICAM-1) ELISA kit, lactate dehydrogenase (LDH) colourimetric assay kit, superoxide dismutase (SOD) assay kit, and antibodies for eNOS and β-actin were purchased from Abcam Co., Ltd. (Shanghai, China). A peroxidase affinipure rabbit anti-mouse IgG was purchased from Jackson ImmunoResearch Laboratories (West Grove, PA, USA). A human vascular cell adhesion molecule 1 (VCAM-1) ELISA kit was obtained from MultiSciences (Lianke) Biotech Co., Ltd. (Hangzhou, China). All other chemicals for cell culture were analytical-grade reagents and standard commercial products from the Beyotime Institute of Biotechnology (Haimen, China) unless otherwise specified.

### 4.2. Cell Culture and Cd Treatment

Human umbilical vein endothelial cells (HUVECs) (China Center for Type Culture Collection, Wuhan, Hubei, China) were cultured in RPMI 1640 medium (HyClone, Logan, UT, USA) supplemented with 10% foetal bovine serum (ScienCell, Carlsbad, CA, USA) and 100 U/mL penicillin–streptomycin solution at 37 °C in a 5% CO_2_ and 95% air-humidified incubator (Thermo Scientific, Waltham, MA, USA). The cells were trypsinized and passaged once reaching 75–90% confluence. For measurements, cells suspended in medium were seeded onto plates at an appropriate density as required by each measurement protocol. The HUVECs were treated with final CdCl_2_ concentrations of 1, 5, or 10 μM in medium containing 10% foetal bovine serum, and control cells were incubated in the same medium containing 10% foetal bovine serum but no CdCl_2_.

### 4.3. Assessments of Cell Growth and Intracellular Cd Exposure

HUVECs were seeded onto 96-well plates at a density of 4 × 10^3^ cells per well for cell viability analysis and onto 12-well plates at 4 × 10^4^ cells per well for measurements of LDH activity, intracellular total protein levels and intracellular Cd concentration. After 24 h of incubation, cells were exposed to 0, 1, 5, or 10 μM of CdCl_2_ (*n* = 8 per group for cell viability; *n* = 6 per group for other assessments) for 48 h. Cell viability, LDH activity, and intracellular total protein concentrations were separately determined using the CCK-8 cell viability assay, LDH colourimetric assay, and enhanced bicinchoninic acid (BCA) protein assay, respectively, according to the manufacturer’s instructions. Intracellular Cd concentrations were measured using a Hitachi Z-2000 polarized Zeeman graphite furnace atomic absorption spectrometer (Hitachi, Tokyo, Japan).

### 4.4. Cell Tube Formation Assay

A 96-well plate was coated with Matrigel (BD Biosciences, Bedford, MA, USA) (50 μL per well). HUVECs were seeded onto six-well plates at a density of 25 × 10^4^ cells per well and incubated for overnight. The cells then were treated with medium containing 10 μM CdCl_2_ or medium without CdCl_2_ for 48 h of incubation. Subsequently, HUVECs were added to the plate coated with Matrigel at a density of 5 × 10^4^ cells per well and incubated for 6 h prior to assessing the tube formation. The formed tubules were observed by a phase-contrast microscope (Nikon, Tokyo, Japan) at 50× magnification, and the number of branch points were manually counted to quantify the tube formation in a randomly-selected field.

### 4.5. Ultrastructural Observations

HUVECs were seeded into 75 cm^2^ culture flasks (Corning, NY, USA) at a density of 5 × 10^5^ viable cells per flask for 24 h of incubation, followed by 48 h of incubation with medium containing 10 μM CdCl_2_ for Cd-exposed samples and medium without CdCl_2_ as a control. Briefly, cells were fixed with 3% glutaraldehyde in phosphate-buffered saline (PBS) at 4 °C for 2 h, and post-fixed in 1% osmium tetroxide for 2 h. The cells were dehydrated by an ethanol gradient and embedded in epoxy resin. Ultra-thin sections were produced using a Leica EM UC7 ultramicrotome (Leica Microsystems, Wetzlar, Germany) and then stained using uranyl acetate and lead citrate. The sections were observed under transmission electron microscopy (TEM) Hitachi H-7650 (Hitachi, Tokyo, Japan).

### 4.6. Measurements of HUVEC Phenotypes

HUVECs were seeded onto 12-well plates at a density of 4 × 10^4^ cells per well for 24 h of incubation and then treated with 0, 1, 5, or 10 μM of CdCl_2_ (*n* = 6 per group) for 48 h prior to performing the following measurements. NO content in the culture supernatant was measured using a nitric oxide colourimetric assay kit to evaluate NO production in HUVECs. To measure the inflammatory response, VCAM-1 and ICAM-1 contents of HUVECs in the culture supernatant were determined using a VCAM-1 ELISA kit and an ICAM-1 ELISA kit, respectively. Oxidative stress was evaluated by measuring the MDA content in culture supernatant and intracellular SOD activity; MDA and SOD were measured using the corresponding assay kits. All measurements were performed according to the instructions provided by the manufacturers.

### 4.7. Western Blot Assay of eNOS

Six-well plates were seeded with HUVECs at a density of 25 × 10^4^ cells per well for overnight incubation. Then HUVECs were incubated with 0, 1, 5, or 10 μM of CdCl_2_ for 48 h, followed by the total protein extraction that was performed in the lysed cells in lysis buffer (containing RIPA:PMSF = 100:1). Briefly, equal amounts of protein were subjected to 10% SDS-polyacrylamide gel electrophoresis, and transferred to a polyvinylidene difluoride membrane sequentially. The membrane was blocked in Tris-buffered saline Tween-20 (TBST) containing 5% non-fat milk for 1 h, then incubated with primary antibodies for total eNOS (1:300 dilution) and β-actin (1:250 dilution) for overnight at 4 °C, followed by an anti-mouse IgG (1:3000 dilution) conjugated to horseradish peroxidase for 1 h at room temperature. After use of a chemiluminescence (Thermo, SuperSignal West Pico, Pierce, Rockford, IL, USA), the final production of protein was recorded using a gel image analysis system (Tanon Co., Ltd., Shanghai, China). The level of eNOS expression was determined by a blot band intensity ratio of total eNOS to the corresponding β-actin.

### 4.8. Cell Sample Collection and Preparation

HUVECs were incubated in 150 cm^2^ culture flasks (Corning, NY, USA) at a density of 1.5 × 10^6^ viable cells per flask for 24 h. Subsequently, the cells were treated with medium containing 10 μM CdCl_2_ in the Cd-exposed group (*n* = 6) and medium without CdCl_2_ in the control group (*n* = 6). After up to 48 h of incubation, the medium was discarded, and the cells were washed 6 times with PBS; the wash solution was centrifuged to collect the cells. Cells in the flask were then scraped off using a cell scraper in the presence of 1.5 mL of methanol. 1.5 mL of cell suspension was combined with the cells isolated from the washing step in centrifuge tubes and stored at −80 °C until further analysis.

A 500-μL volume of thawed cell suspension was transferred into 2-mL centrifuge tubes, and then 30 μL of L-2-chlorophenylalanine (0.1 mg/mL stock in dH_2_O) was added as an internal standard. The solution was vortexed for 10 s followed by 3 ultrasonic vibrations for 5 min each time; the solution was subsequently centrifuged at 12,000 rpm for 15 min at 4 °C. Then, 0.4 mL of the supernatant was transferred into a 2-mL silylated glass vial and dried in a vacuum concentrator at 30 °C for approximately 1.5 h. An 80-μL volume of methoxymethyl amine salt (dissolved in pyridine, final concentration of 20 mg/mL) was added to the dried extracts and gently mixed; the mixture was then incubated in an oven at 37 °C for 2 h. In addition, 100 μL of bistrifluoroacetamide (containing 1% TCMS, *v*/*v*) was added to each mixture and incubated at 70 °C for 1 h. Finally, the mixture was cooled to room temperature, followed by adding 10 μL of FAMEs (standard mixture of fatty acid methyl esters, C8-C16: 1 mg/mL; C18-C30: 0.5 mg/mL in chloroform) for GC-TOF/MS analysis.

### 4.9. GC-TOF/MS Analysis

The GC-TOF/MS analysis was performed using an Agilent 7890 gas chromatograph system coupled with a Pegasus HT time-of-flight mass spectrometer (LECO Chroma TOF PEGASUS HT, LECO, Saint Joseph, MI, USA), which utilized a Rxi-5Sil MS capillary column coated with 5% diphenyl cross-linked with 95% dimethylpolysiloxane (30 m × 250 μm inner diameter, 0.25 μm film thickness; Restek, Bellefonte, PA, USA). A 1-μL aliquot of the analyte was injected in the splitless mode, and helium was used as the carrier gas, with a 3 mL/min front inlet purge flow and 1 mL/min gas flow rate through the column. The initial temperature of the column was maintained at 50 °C for 1 min; then, it was raised to 330 °C at a rate of 10 °C/min and maintained for 5 min. The temperatures for the injection, transfer line and ion source were 280, 280, and 220 °C, respectively. The mass spectrometry data were acquired using a mass-to-charge ratio (*m*/*z*) range of 85–600 at a rate of 20 spectra per second after a solvent delay of 366 s in the full-scan mode with electron impact ionization at 70 eV. The stability of the injected sample was tested by evaluating the retention time (RT) of the internal standard.

### 4.10. Data Processing and Statistical Analysis

Raw data were acquired using Chroma TOF 4.3X software (LECO Corporation, Saint Joseph, MI, USA) and the LECO-Fiehn Rtx5 database after a standard process including the extraction of raw peaks, data baseline filtering and calibration, peak alignment, deconvolution analysis, peak identification, and peak area integration. The RT index (RI) method was used in the peak identification, and the RI tolerance was 5000. Missing values in the raw data were filled up by a simulation method with half of the minimum value. The raw peaks were retained using the interquartile range denoising method, and then, the corresponding data were processed using an internal standard normalization method. For compound identification, the LECO/Fiehn Metabolomics Library was used to evaluate the accuracy based on a similarity value. A similarity value greater than 700 indicated reliable metabolite identification. When the similarity value was less than 200, “analyte” was used as the compound name. When the similarity value was between 200 and 700 indicated that the compound is a putative annotation. Endogenous metabolites were further verified by searching the Kyoto Encyclopedia of Genes and Genomes (KEGG) database (http://www.genome.jp/kegg) [64] and the Human Metabolome Database [65].

The resultant three-dimensional data, including the peak number (RT and *m*/*z* pairs), sample name (observation), and normalized peak area (variable), were exported to the SIMCA 13.0 software package (Umetrics, Umea, Sweden) for principal component analysis (PCA), partial least-squares discriminant analysis (PLS-DA), and orthogonal projections to latent structures discriminant analysis (OPLS-DA). PCA was used to profile the distribution of raw data. A supervised PLS-DA was performed to estimate the robustness and the predictive ability of models using seven-fold cross-validation and permutation tests. To obtain a higher level of group separation and a better understanding of variables responsible for classification, OPLS-DA was used to discriminate the different metabolites between the Cd-exposed group and the control group. The significantly different metabolites were selected based on a statistical value greater than 1.0 in the first principal component of variable importance in the projection (VIP) and a *p* value of less than 0.05 by Student’s *t*-test between two groups.

In addition, the relevant metabolic pathway was matched with each differential metabolite by searching the KEGG database. The differential metabolites were further imported into the online MetaboAnalyst [66] for pathway analysis with integrated enrichment analysis and pathway topology analysis [67]. *Homo sapiens* (human) was selected as the pathway library, and a hypergeometric test and relative-betweenness centrality were used for the pathway enrichment analysis and the pathway topology analysis, respectively.

## 5. Conclusions

In summary, using a non-targeted metabolomics approach, we identified 24 endogenous metabolites and seven metabolic pathways that were significantly disturbed by non-toxic Cd exposure in vascular endothelial cells. Our findings from the current metabolic profiling further suggest that the metabolic alterations linked to TCA cycle dysfunction, glycosylation of the HBP, and compensatory responses to genomic instability and energy deficiency may be generally associated with decreased endothelial NO production as well as a proinflammatory state and non-significant oxidative stress. The present study may help to increase an understanding of the pathogenesis of non-toxic Cd-induced vascular endothelial dysfunction from a metabolic standpoint.

## Figures and Tables

**Table 1:** Significantly different metabolites in HUVECs between the Cd-exposed and control groups.

Metabolite	Similarity	RT (minutes)	Mass	VIP	*p* Value	Fold Change	Classification
ornithine	973	16.88	142	1.66	0.003	1.81	amino acids
glycine	957	8.15	102	1.41	0.021	2.60	amino acids
citrulline	926	16.95	157	1.27	0.043	1.55	amino acids
l-cysteine	920	13.95	220	1.41	0.032	3.03	amino acids
trans-4-hydroxy-l-proline	889	12.97	158	1.71	0.007	2.99	amino acids
aspartic acid	778	12.34	160	1.28	0.042	2.64	amino acids
l-homoserine	775	12.63	218	1.42	0.039	204.37	amino acids
uracil	938	11.18	99	1.45	0.034	7.38	pyrimidines
thymine	822	12.02	255	1.50	0.027	7.98	pyrimidines
orotic acid	804	16.10	254	1.41	0.039	10.53	pyrimidine derivatives
guanine	883	19.93	352	1.43	0.037	9.16	purines
hypoxanthine	833	16.83	265	1.49	0.028	6.77	purine derivatives
ribose	895	15.34	103	1.44	0.035	5.74	monosaccharides
glyceric acid	864	11.06	189	1.49	0.024	2.98	sugar acids and derivatives
*N*-acetylmannosamine	701	19.44	202	1.44	0.036	169.09	monosaccharides
pyruvic acid	915	7.11	174	1.40	0.042	11.33	2-oxocarboxylic acids
glycolic acid	865	7.49	147	1.37	0.026	1.77	hydroxy acids and derivatives
ribonolactone	802	15.41	117	1.52	0.011	1.75	lactones
ethanolamine	886	10.22	174	1.44	0.037	18.39	alkanolamines
pelargonic acid	728	11.54	117	1.40	0.022	1.69	fatty acids
l-malic acid	966	13.11	147	1.67	0.003	0.30	hydroxycarboxylic acids
fumaric acid	923	11.36	245	1.28	0.042	0.70	dicarboxylic acids and derivatives
citric acid	911	16.88	273	1.57	0.007	0.21	tricarboxylic acids and derivatives
3-phosphoglyceric acid	916	16.74	299	1.33	0.033	0.32	sugar acids and derivatives

**Table 2:** Differential metabolites involved in the disturbed metabolic pathways in HUVECs.

Metabolic Pathways	Differential Metabolites (Fold Change)					
Glycine, Serine and Threonine	glyceric acid (↑)	glycine (↑)	aspartic acid (↑)	l-homoserine (↑)	l-cysteine (↑)	pyruvic acid (↑)
TCA Cycle	pyruvic acid (↑)	l-malic acid (↓)	citric acid (↓)	fumaric acid (↓)		
Arginine and Proline	ornithine (↑)	aspartic acid (↑)	citrulline (↑)	trans-4-hydroxy-l-proline (↑)	fumaric acid (↓)	pyruvic acid (↑)
Alanine, Aspartate and Glutamate	aspartic acid (↑)	pyruvic acid (↑)	fumaric acid (↓)			
Cysteine and Methionine	l-cysteine (↑)	l-homoserine (↑)	aspartic acid (↑)	pyruvic acid (↑)		
Pyrimidine	uracil (↑)	thymine (↑)	orotic acid (↑)			
Pyruvate	pyruvic acid (↑)	l-malic acid (↓)				

The mark “↑” represents a fold change value greater than 1; and “↓” represents a fold change value less than 1.
